# Modelling Strategies for Reinforced Concrete Elements under Corrosion Degradation

**DOI:** 10.3390/ma15134601

**Published:** 2022-06-30

**Authors:** Stefania Imperatore

**Affiliations:** Department of Civil Engineering, “Niccolò Cusano” University, Via Don Carlo Gnocchi, 3, 00166 Rome, Italy; stefania.imperatore@unicusano.it

**Keywords:** reinforced concrete, corrosion, load-carrying capacity, local ductility, modelling strategies

## Abstract

Aging and corrosion of reinforced concrete structures (RCS) is becoming a global problem, thus proper procedures for simulating the structural performance of corroded RCS should be assessed. Among the main corrosion effects, concrete cover cracking and reinforcement cross-section reduction may influence the materials’ constitutive laws, moreover the confinement contribution and the lateral instability of the longitudinal rebars can be modified. In the present paper, the predictive models available in the scientific literature to assess the materials’ mechanical properties of corroded RCS are recalled and employed into a novel model to derive the theoretical moment–curvature relationships for the cross-section of square and rectangular corroded reinforced concrete elements. The model accounts for cover spalling, buckling of longitudinal reinforcing bars, reduction in confinement pressures, reduction in concrete constitutive law due to the concrete cracking induced by rust formation and decay of mechanical properties for corroded reinforcements. The obtained results are compared with the classical simplified models for corroded RCS, highlighting that buckling and confinement variations cannot be disregarded into a reliable modelling strategy, especially when local ductility plays a key role in the performed investigations.

## 1. Introduction

Most existing reinforced concrete (RC) structures in industrialized countries have at least 40 years [1,2] and are characterized by obsolete design methodologies, disregarding appropriate seismic principles [3,4] and durability issues [5,6,7,8]. Since corrosion degradation is becoming a problem of global relevance, due to the aging coupled to continuous environment and climate changes, the seismic assessment of corroded RC structures is shaping up to be one of the new frontiers of research. To this aim, a proper evaluation of the residual ductility of corroded RC members plays a fundamental rule. Moreover, the simulation of the seismic performance of complex structural systems requires the assessment of optimized modelling strategies, catching the effective behavior of RC members subjected to corrosion degradation.

Under corrosion, RC elements progressively decrease their structural capacity due to: (i) concrete cracking and the decay of its compressive strength [9,10], (ii) modification of the bond-slip law between reinforcement and concrete [11,12,13,14], (iii) variation in the tension-stiffening effects [15,16], and (iv) the cross-section reduction of steel reinforcements and the decay of their mechanical properties [17].

Consequently, the whole structure under seismic actions may have a performance worse than the estimated one. Nowadays, the assessment of corroded RC structures is mainly modeled by considering the steel and (eventually) the concrete deterioration [18,19,20,21]. Rarely, the bond-slip law as well as the buckling effects are considered [22,23]. 

The main object of the present research is to propose a novel simplified model to assess theoretical moment–curvature relationships for rectangular/square corroded reinforced concrete elements. The model accounts for the corrosion effects on: (i) concrete cover cracking, (ii) concrete confinement, (iii) steel constitutive law, and (iv) lateral instability of the longitudinal reinforcement. Moreover, a parametric analysis is carried out to evaluate the most effective modeling strategy for corroded reinforced concrete structures. The obtained results, compared to the ones derived for classical simplified models, allow the assessment of the optimal modelling strategy which assesses the sectional response of corroded structural elements in RCS. 

## 2. Constitutive Laws of Materials after Corrosion Degradation

A sensible reduction in the structural capacity of corroded RC elements is observed for corrosion levels higher than 10% in mass loss [24,25]. At this stage, the bond strength can be considered completely loss [14,16], and relevant variations in the reinforcement mechanical properties can be observed in case of pitting corrosion [17]. Moreover, the concrete constitutive law reduces due to the cracking caused by the oxides’ expansion [9,10,26,27,28]. In the following sections, the main constitutive relationships simulating both the corroded reinforcement and the cracked concrete are recalled. Among all, proper decay laws are selected for their feasibility in simulating some experimental results [29,30].

### 2.1. Influence of Corrosion Cracking on Concrete Constitutive Law

Since the corrosion products have a density lower than the sound steel, a radial internal pressure stresses the concrete when the reinforcements corrode. Once the tangential stresses caused by the oxides’ expansion exceed the concrete tensile strength, cracks progressively grow in the concrete cover and enlarge, increasing the corrosion level [31]. 

According to [32], the tensile strains in the transverse direction reduce the concrete compressive strength. From this perspective, a reduction in the mechanical properties for concrete cracked by corrosion is expected and the residual strength of the concrete cover can be theoretically estimated as [9]:

(1) $$f_{c,\;\;\;corr}=\frac{1}{1+k\frac{\varepsilon_{ave}}{\varepsilon_{c0}}}\cdot f_{c}$$

where *k* is a coefficient related to bar roughness and diameter, *ε_c_*_0_ the strain at the peak compressive stress *f_c_*; and *ε_ave_* the smeared tensile strain in the cracked concrete defined as:

(2) $$\varepsilon_{ave}=\frac{\pi nD\left[\rho_{rust}-1\right]\left[1-0.1\sqrt{100-CL}\right]}{b_{0}}$$

where *n* represents the number of corroded bars, *ρ_rust_* is the ratio of volumetric expansion of the oxides with respect to the virgin material, *D* is the initial reinforcement diameter, *b*_0_ is the section width in the virgin state and *CL* is the corrosion level expressed in mass loss.

The experimental tests carried out on corroded specimens by [10] allow the definition of the residual strength of the cracked concrete as:

(3) $$f_{c,\;\;\;corr}=\left(1-\lambda \right)\cdot f_{c}$$

being

(4) $$\begin{array}{cc} \lambda =2.720\cdot CL-1.980 & for\;\;water-to-cement\;ratio\;equal\;to\;0.40\\ \lambda =2.288\cdot CL-1.733 & for\;\;water-to-cement\;ratio\;equal\;to\;0.45\\ \lambda =2.576\cdot CL-1.876 & for\;\;water-to-cement\;ratio\;equal\;to\;0.50 \end{array}$$

Instead, according to the experimental campaign performed on concrete cores extracted by corroded piers [26], the regression coefficient estimated for the residual strength of the cracked concrete are:

$$\begin{array}{cc} \lambda =0.773\cdot CL & if\;the\;average\;values\;of\;the\;concrete\;strength\;are\;considered\\ \lambda =1.121\cdot CL & if\;the\;5\%\;values\;of\;the\;concrete\;strength\;are\;considered \end{array}$$

Moreover, in [26], the peak strain of the concrete cover damaged by the steel reinforcement corrosion is suggested:

(5) $$\varepsilon_{c\_peak,\;\;\;corr}=\left(1-Q\right)\cdot \varepsilon_{c\_peak}\;\begin{array}{cc} Q=0.549\cdot CL & if\;the\;average\;values\;of\;the\;concrete\;strength\;are\;considered\\ Q=0.841\cdot CL & if\;the\;5\%\;values\;of\;the\;concrete\;strength\;are\;considered \end{array}$$

Finally, the classical Mander’s model [33] is adapted by [27,28] for estimating the concrete strength confined by corroded stirrups. According to [27], the mechanical properties of confined concrete for corroded RC columns can be evaluated as:

(6) $$f_{cc}^{corr}=\left(1-\alpha \cdot CL\right)\cdot f_{c0}\cdot \left(2.254\cdot \sqrt{1+\frac{7.94\cdot f_{l}^{corr}}{f_{c0}}}-\frac{2\cdot f_{l}^{corr}}{f_{c0}}-1.254\right)\;\varepsilon_{cc}^{corr}=\left(1-\beta \cdot CL\right)\cdot \varepsilon_{c0}\cdot \left[1+5\left(\frac{f_{cc}^{corr}}{f_{c0}}-1\right)\right]\;\varepsilon_{cu}^{corr}=0.004+\left(1-CL\right)\cdot \frac{1.4\cdot \rho_{s}\cdot f_{yh}^{corr}\cdot \varepsilon_{su}^{corr}}{f_{cc}^{corr}}$$

where *α* is the yield strength reduction factor for corroded transverse reinforcement (equal to: 0.19 for square confined section with single hoops configuration, 0.40 for square sections with double hoops configuration and 0.51 for circular confined section with spiral hoops), *β* is a strain correction coefficient (equal to: 0.49 for square confined section with single hoops configuration, 1.29 for square sections with double hoops configuration and 0.28 for circular confined section with spiral hoops). The parameter $f_{l}^{corr}$ is the effective lateral confining stress, defined as:

(7) $$f_{l}^{corr}=\frac{1}{2}\cdot k_{e}\cdot \left(1-CL\right)\cdot \rho_{s}\cdot f_{yh}^{corr}$$

where *k_e_* is the confinement effectiveness coefficient defined according to [33].

Similarly, in [28] the following relationships are suggested to estimate the mechanical properties of confined concrete in corroded circular RC columns:

(8) $$f_{cc}^{corr}=f_{c0}\cdot \left(2.254\cdot \sqrt{1+\frac{7.94\cdot f_{l}^{corr}}{f_{c0}}}-\frac{2\cdot f_{l}^{corr}}{f_{c0}}-1.254\right)\;\varepsilon_{cc}^{corr}=\varepsilon_{c0}\cdot \left[1+5\left(\frac{f_{cc}^{corr}}{f_{c0}}-1\right)\right]\;\varepsilon_{cu}^{corr}=0.004+\frac{1.4\cdot \rho_{s}\cdot f_{yh}^{corr}\cdot \varepsilon_{sm}^{corr}}{f_{cc}^{corr}}$$

where the effective lateral confining stress is:

(9) $$f_{l}^{corr}=\frac{2\cdot f_{yh}^{corr}\cdot A_{sp}}{d_{s}\cdot s}\cdot \frac{1-\frac{s}{2d_{s}}}{1-\frac{A_{sl}}{A_{c}}}$$

where *A_sp_* is the sectional area of transverse reinforcement, *d_s_* is the diameter of spiral reinforcement, *s* is the vertical spacing between spirals, *A_sl_* is the cross-section of longitudinal reinforcement and *A_c_* is the concrete core sectional area.

In the previous relationships (Equations (6)–(8)):

- $f_{cc}^{corr}$, $\varepsilon_{cc}^{corr}$ and $\varepsilon_{cu}^{corr}$ respectively represent the compressive strength, the strain at compressive strength and the ultimate strain of confined concrete damaged by corrosion;
- *ε_c_*_0_ is axial strain of unconfined concrete corresponding to maximum unconfined stress. *ε_c_*_0_ = 0.002 can be assumed when test data are not available;
- $f_{yh}^{corr}$ and $\varepsilon_{su}^{corr}$ are the yield stress and the ultimate strain of corroded transverse reinforcements, respectively;
- *ρ_s_* is the ratio of volume of transverse reinforcement respect to the volume of confined concrete core;
- *f_c_*_0_ is the compressive strength of unconfined concrete.

### 2.2. The Monotonic Mechanical Properties of Corroded Steel Reinforcements

**Figure 1:**
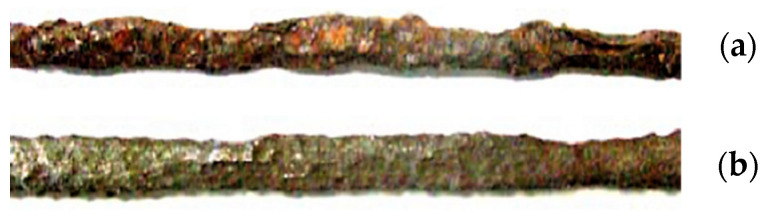
Comparison between: (**a**) natural and (**b**) artificial corrosion.

The shape of naturally corroded steel reinforcement is well simulated by the artificial corrosion (Figure 1). Therefore, if the process is not excessively accelerated, similar mechanical properties are expected in artificially and naturally corroded bars. The literature’s experimental outcomes highlight a reduction in the mechanical properties increasing the corrosion level, allowing the derivation of different degradation laws, a useful tool to assess the structural performance of corroded RC structures. In detail, linear relationships relate strength and corrosion level *CL*, while the ultimate deformation can follow a linear or an exponential trend. A complete review on the degradation laws of corroded reinforcements can be found in [17].

The state-of-the-art on the monotonic behavior under compression highlighted that the buckling effects become relevant for slenderness ratios (λ = *l/ϕ*) higher than 10 [34,35,36,37]. Only two models are available in the scientific literature concerning the mechanical performance in compression of corroded reinforcement, respectively, developed for uniform [35] and pitting corrosion [36,37]. Concerning the uniform corrosion [35], the reduction in the peak stress in compression follows a decay law depending on both the elastic Eulerian buckling strength and the reduction in the yielding stresses in tension. The post-buckling behavior instead depends on both the slenderness ratio and the cinematic response of the corroded steel rebars. Concerning the pitting corrosion, instead, the statistical regression of the experimental results from [36,37] governs the decay law for the peak strength, while the post-yield buckling response is derived modifying to the classical model by [38] to account for the corrosion degradation.

### 2.3. Choice of the Constitutive Law for Simulating Corroded Reinforced Concrete Elements Using Experimental Data

With the main aim to define a proper modelling approach for corroded RC members, the structural performance of the uncorroded and corroded specimens has been simulated in OpenSees according to a distributed plasticity approach. To this aim, a monotonic pushover analysis based on a fiber-section model has been implemented. The uniaxial Hysteretic material model has been adopted to simulate the performance of the steel reinforcements in both tension and compression. In the latter case, the Dhakal–Maekawa buckling model [38] is considered for the sound specimen. The Concrete02 material available in OpenSees has been instead employed to model the concrete stress–strain law [33]. 

The concrete cracking and the variation of the reinforcement mechanical properties are introduced in the numerical model to account for the corrosion effects. According to [39], only the concrete cover should be regarded as cracked and the deterioration of the concrete material properties due to the presence of the cracks is evaluated by adopting the approach proposed in [9]. The corrosion influence on the constitutive law of the confined concrete is considered by means of the relationships proposed in [27], based on the classical Mander’s model [33]. Concerning the reinforcement material properties, the degradation law for pitting corrosion proposed in [17] and in [36] for the tensile and the compressive behavior are introduced, respectively.

**Figure 2:**
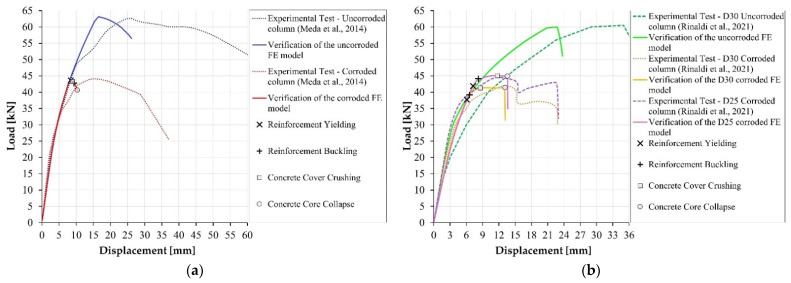
Simulation of corroded reinforced concrete elements by using a numerical model. Verification of the Finite Element Model: (**a**) comparison with the experimental campaign presented in [29] (readaptation of original images); (**b**) experimental campaign presented in [30] (readaptation of original images).

The numerical model is validated by considering the experimental tests performed in [29,30], as shown in Figure 2. According to the obtained results, the constitutive laws proposed in [9,27,36,40] can be chosen for simulating the performance of corroded reinforced concrete elements, provided that the phenomena like the fixed-end rotation or premature slip occurrence are avoided. This is the case of the specimens tested in [29], characterized by a construction joint between the column and the foundation, or of the uncorroded specimen tested in [30], subjected to excessive slips between concrete and reinforcement due to the inhomogeneous mixture casted. In both cases, the specimens’ deformability cannot be perfectly simulated if the additional zero-length element suggested in [39] is disregarded in the numerical model.

## 3. Local Behavior of Corroded Reinforced Concrete Elements

**Figure 3:**
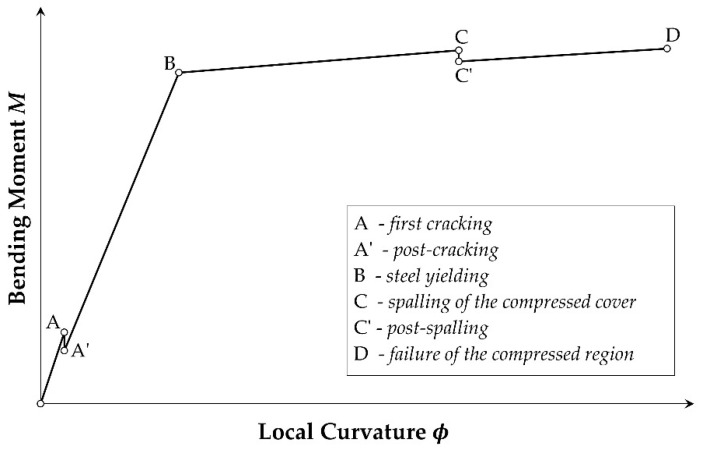
Typical moment–curvature diagram represented considering the buckling effects.

The local behavior of reinforced concrete elements can be evaluated by means of a moment–local curvature relationship able to account for both the confinement and the buckling influence. The typical moment–curvature diagram is shown in Figure 3: point A represents the first cracking of the section; point B represents the yielding of the reinforcement in tension; point C defines the concrete spalling, and point D defines the element collapse. Points A’ and C’ describe the element behavior immediately after the concrete cracking and the spalling, respectively.

The neutral axis position *x_n_* is evaluated imposing the equation of equilibrium to the axial load *N*:

(10) $$\begin{array}{c} N=\int_{x_{n}-\delta }^{x_{n}}\left(b-2\delta \right)\sigma_{cnc}\left(\varepsilon_{c}\left(x_{n}\right)\right)dx+\int_{0}^{x_{n}}2\delta \sigma_{cnc}\left(\varepsilon_{c}\left(x_{n}\right)\right)dx+\\ \int_{0}^{x_{n}-\delta }\left(b-2\delta \right)\sigma_{cc}\left(\varepsilon_{c}\left(x_{n}\right)\right)dx+A_{sc}\sigma_{sc}\left(\varepsilon_{c}\left(x_{n}-\delta \right),\lambda \right)-A_{st\;}\sigma_{st}\left(\varepsilon_{c}\left(d-x_{n}\right)\right) \end{array}$$

where *b* is the section width, *d* is the effective section depth, *δ* is the section concrete cover, *x_n_* is the neutral axis, *x* is the depth of the generalized concrete fiber, *A_sc_* and *A_st_* represent the sound area of the reinforcement in the compressive and tensile region, respectively; *σ_cc_* and *σ_cnc_* are the constitutive laws of the confined and not confined concrete, accounting for the effects of both the concrete cracking due to the oxides’ expansion and the stirrups’ corrosion; *σ_st_* and *σ_sc_* are the ones of the tensed and the compressed corroded reinforcement, respectively (the latter depends on the slenderness *λ= s_st_/ϕ,* defined as the ratio between the stirrups spacing *s_st_* and the longitudinal reinforcement diameter *ϕ*).

Knowing the neutral axis position and consequently the relative curvature *φ*, the bending moment *M* is given by:

(11) $$\begin{array}{c} M-N\left(\frac{h}{2}-x_{n}\right)=\int_{x_{n}-\delta }^{x_{n}}\left(b-2\delta \right)\sigma_{cnc}\left(\varepsilon_{c}\left(x_{n}\right)\right)xdx+\int_{0}^{x_{n}}2\delta \sigma_{cnc}\left(\varepsilon_{c}\left(x_{n}\right)\right)xdx\\ +\int_{0}^{x_{n}-\delta }\left(b-2\delta \right)\sigma_{cc}\left(\varepsilon_{c}\left(x_{n}\right)\right)xdx+A_{sc}\sigma_{sc}\left(\varepsilon_{c}\left(x_{n}-\delta \right),\lambda \right)\left(x_{n}-\delta \right)\\ +A_{st}\sigma_{st}\left(\varepsilon_{c}\left(d-x_{n}\right)\right)\left(d-x_{n}\right)\cdot \end{array}$$

In the cracking stage (point A), the contribution of the concrete in tension is considered and the section is characterized by a curvature $\phi_{cr}=\frac{\varepsilon_{ct}}{h-x_{A}}$, as *x_A_* the neutral axis at the cover cracking; subsequently, the sectional behavior at the post-cracking stage (point A’) is evaluated imposing a curvature $\phi =\phi_{cr}$ into the hypothesis that the cracked section doesn’t work. Similarly, the curvature at the yielding point B is evaluated as $\phi_{y}=\frac{\varepsilon_{sy}}{h-x_{B}}$, being *x_B_* the neutral axis and ε_sy_ the yielding strength of the corroded reinforcement. When the concrete starts to crush, a check on the concrete strains is necessary. If the maximum strain of both concrete and reinforcement is lower than the materials’ ultimate strain at the spalling stage, a collapse without spalling can occur, and the moment–curvature relationship can be limited to the point C, defined by evaluating the ultimate bending moment *M_U_ = M_C_* and the ultimate local curvature *ϕ_U_* = *ϕ_C_*. This means that the corroded section breaks down when the concrete cover reaches an ultimate strain larger than the conventional 3.5‰ (estimated proportionally to the concrete strength reduction defined in [9]) or, alternatively, when the tensed reinforcement reaches the ultimate strain ε_su_ defined according to the relationship proposed in [40]. In both cases, the local ductility is conventionally evaluated as $\mu =\frac{\phi_{C}}{\phi_{y}}$. 

If, instead, the spalling starts (point C’), the sectional collapse occurs at the concrete core crushing. The latter condition is evaluated according to the constitutive law defined in [27] for concrete confined by corroded stirrups. In this condition, the compressed cover cannot work anymore, the section is in the post-spalling stage (C’-D) and the section is characterized by a curvature $\phi_{sp}=\frac{\varepsilon_{cmax}}{x_{C}}$, with *x_C_* the neutral axis at the cover spalling. In this condition, the local ductility can be estimated as $\mu =\frac{\phi_{sp}}{\phi_{y}}$ while the ultimate bending moment becomes *M_U_* = *M_D_*.

On the theoretical basis previously described, a parametrical analysis has been carried out, with the main aim to assess the better modeling strategy for the corroded reinforced concrete elements. As a reference, a typical column designed about 50 years ago and characterized by a rectangular section 30 × 50 cm^2^ is considered. The materials and reinforcement arrangement are defined according to the provision of the Decree of Italian Ministry of 30 May 1972 that introduced the use of ribbed bars for RC structures [41]. In detail, the maximum or minimum stirrups spacing (affecting both the slenderness ratio *λ* and the concrete confinement level) as well as a different mechanical percentage of longitudinal reinforcement *ω* (maximum, minimum, average) are considered. As usual, the mechanical percentage of longitudinal reinforcement *ω* is defined as $\omega =\frac{A_{s}\cdot f_{y0}}{b\cdot d\cdot f_{c0}}$, where *f_c_*_0_ and *f_y_*_0_ are the concrete compressive strength and the yielding strength of the uncorroded section, respectively. On the reference element, different corrosion levels are introduced according to the modelling strategy applied in Section 2.3. Moreover, the corrosion effects on the performance of confined concrete are accounted for by adopting the approach proposed in [27].

With the main aim to evaluate the most performing modeling strategy, simplified approaches are finally implemented, and their results are compared to the reference one.

### 3.1. Load-Carrying Capacity of Corroded Reinforced Concrete Elements

**Figure 4:**
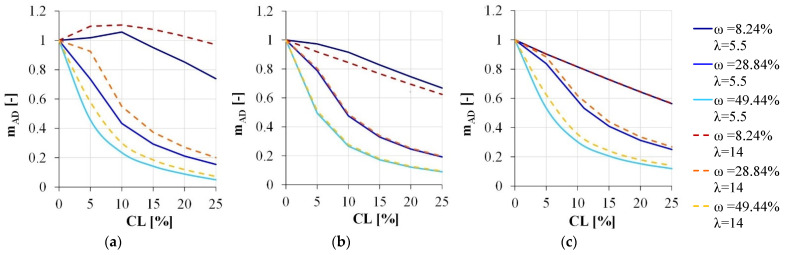
Load-carrying capacity reduction due to the corrosion degradation: (**a**) ν = 25.65%, (**b**) ν = 15.39%, (**c**) ν = 5.13%.

The performance of the reference element is firstly analyzed in terms of load-capacity reduction. At this aim, the nondimensional ultimate bending moment after corrosion (**m_AD_**) was introduced and evaluated as $m_{AD}=\frac{M_{U,corr}}{M_{U,0}}$, being *M_U,corr_* and *M_U,0_* the ultimate bending moment of the corroded and sound section, respectively. In Figure 4, the variation of **m_AD_** is plotted as a function of the corrosion level (*CL*) expressed as $CL=\frac{A_{st,0}-A_{st,corr}}{A_{s,0}}$, where *A_st,_*_0_ and *A_st,corr_* are the reinforcement cross section in the sound and corroded condition, respectively. For the sake of simplicity, in the parametric analysis both stirrups and longitudinal reinforcements are assumed as affected by the same corrosion amount. According to previous results obtained by the author in more simplified approaches [42,43], increasing the corrosion level, the load-carrying capacity typically reduces, with some exceptions. In fact, for a low corrosion level, the structural response of the most compressed section (ν = 25.65%, being $\nu =\frac{N_{rd}}{b\cdot d\cdot f_{c0}}$ where *N_rd_* is the design axial load according to [41]) is always governed by buckling and the spalling phenomena. Therefore, due to the progressive reduction in the neutral axis, an initial increment of the load-carrying capacity can be observed when the flexural contribution of the axial load becomes relevant on the internal stresses. Such an effect is more consistent in case of large stirrups spacing, due to the lower level of the internal forces caused by the reduced confinement and by the buckling occurrence.

To evaluate the effectiveness of simplified models in catching the sectional response of the corroded section, the nondimensional ultimate bending moment after corrosion **m_AD_** (hereinafter called **m_AD reference_**) is compared with the one estimated by disregarding: (i) only the buckling occurrence (**m_AD no buckling_**), (ii) only the concrete cover (**m_AD no cover_**), (iii) both the concrete cover and the buckling occurrence (**m_AD no cover+buckling_**). Since the buckling occurrence mostly affects the plastic hinge zone of columns subjected to seismic excitation, only the cases characterized by a maximum (ν = 25.65%) or intermediate superimposed axial load (ν = 15.39%) are considered.

**Figure 5:**
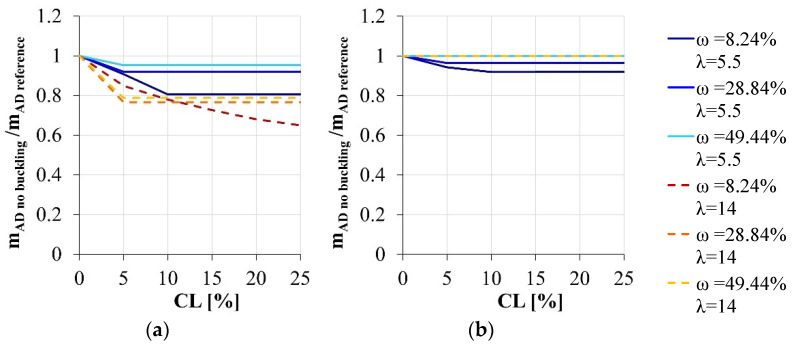
Load-carrying capacity variation due to the corrosion degradation: comparison between the simplified model disregarding the buckling effects and the reference section. (**a**) ν = 25.65%, (**b**) ν = 15.39%.

If the buckling effects are disregarded in the modelling approach (Figure 5), the simplified model can underestimate the effective sectional load-carrying capacity, with an error generally lower than 25%. The only exception is the case of the compressed section with the larger stirrups spacing and the lower longitudinal reinforcement amount, for which the two modeling approaches always bring to different failure modes increasing the corrosion level.

**Figure 6:**
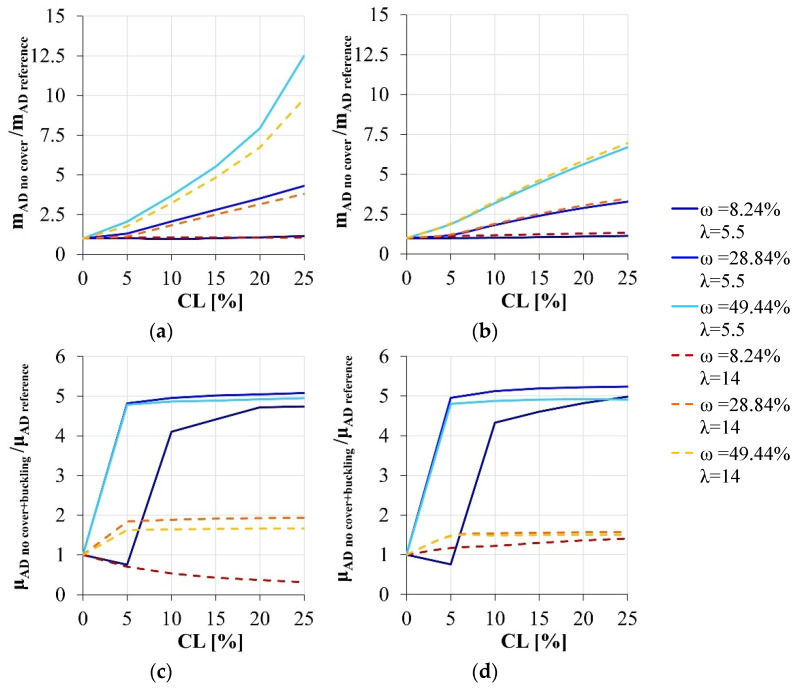
Load-carrying capacity variation due to the corrosion degradation: comparison between the simplified models and the reference section. (**a**) ν = 25.65%, section modeled disregarding only the concrete cover vs. reference section. (**b**) ν = 15.39%, section modeled disregarding only the concrete cover vs. reference section. (**c**) ν = 25.65%, section modeled disregarding both the concrete cover and the buckling effects vs. reference section. (**d**) ν = 15.39%, section modeled disregarding both the concrete cover and the buckling effects vs. reference section.

Finally, the classical simplified models are considered, disregarding the concrete cover and eventually the influence of the lateral instability of the longitudinal reinforcement (Figure 6). In detail, disregarding only the concrete cover, a significant overestimation of the effective load-carrying capacity can be attained by adopting the simplified models, that progressively reduces increasing the superimposed axial load. Instead, the error of the simplified approach become reasonable if both the instability of the compressed reinforcement and the contribution of the concrete cover are disregarded: an underestimation of the real response can be observed that amount to about the 10% if the superimposed axial load is 30% of the maximum sectional capacity in compression.

### 3.2. Local Ductility of Corroded Reinforced Concrete Elements

Once assessed the corrosion influence on the sectional load-carrying capacity, the response in terms of local ductility is investigated. At this aim, the nondimensional local ductility after corrosion (**μ_AD_**) is introduced, defined as $\mu_{AD}=\frac{\mu_{U,corr}}{\mu_{U,0}}$ with *μ_U,corr_* and *μ_U,_*_0_ the local ductility of the corroded and sound section, respectively. 

**Figure 7:**
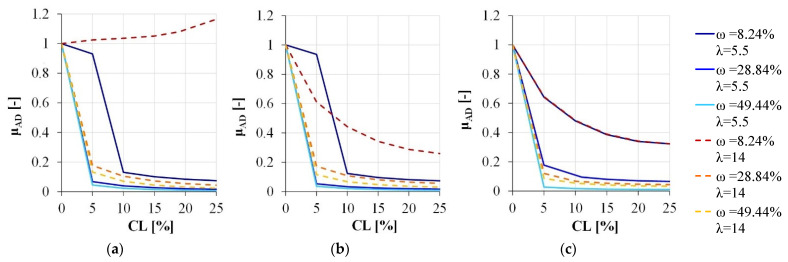
Local ductility variation due to the corrosion degradation: (**a**) ν = 25.65%, (**b**) ν = 15.39%, (**c**) ν = 5.13%.

Considering the reference section, a sharp reduction in local ductility can be observed increasing the corrosion level (Figure 7). In detail, **μ_AD_** takes the same trend for both the high and the medium axial load level. The only exception regards the section with the larger stirrups spacing and the lower longitudinal reinforcement amount, which ductility increases in the presence of a high compression. This is due to the failure mode characterizing the section, governed by concrete spalling and reinforcement buckling for all considered corrosion level. In all other cases, the stirrups’ influence on both the buckling occurrence and the confinement effects appears negligible for corrosion levels higher than 15%.

Finally, the effectiveness of the simplified models in simulating the response of the corroded section in terms of local ductility is investigated considers only the cases characterized by ν = 25.65% or ν = 15.39%, according to the choice made in the previous section. At this aim, the nondimensional local ductility after corrosion (hereinafter called **μ_AD reference_**) is compared with the one estimated by disregarding: (i) only the buckling occurrence (**μ_AD no buckling_**), (ii) only the concrete cover (**μ_AD no cover_**), (iii) both the concrete cover and the buckling occurrence (**μ_AD no cover+buckling_**). 

**Figure 8:**
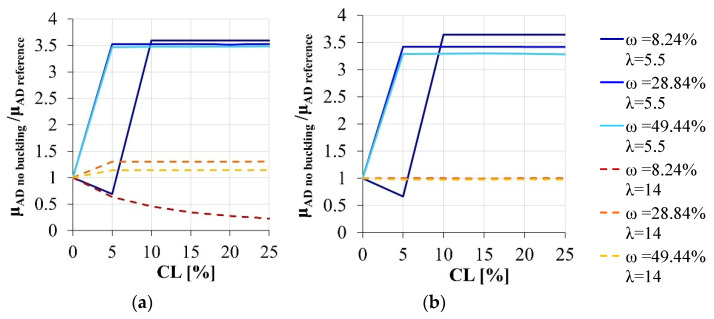
Local ductility variation due to the corrosion degradation: comparison between the simplified model disregarding the buckling effects and the reference section. (**a**) ν = 25.65%, (**b**) ν = 15.39%.

If the stirrups’ spacing is sufficiently large, as in the case of old existing RC structures, the omission of the buckling effects does not cause errors for an intermediate level of the superimposed axial load (Figure 8). An overestimation of the effective local ductility can be instead observed for the high compressive level, excepting for the section characterized by the lower reinforcement amount, due to its peculiar failure mode. On the contrary, the buckling effects cannot be disregarded in the case of reduced stirrups’ spacing.

**Figure 9:**
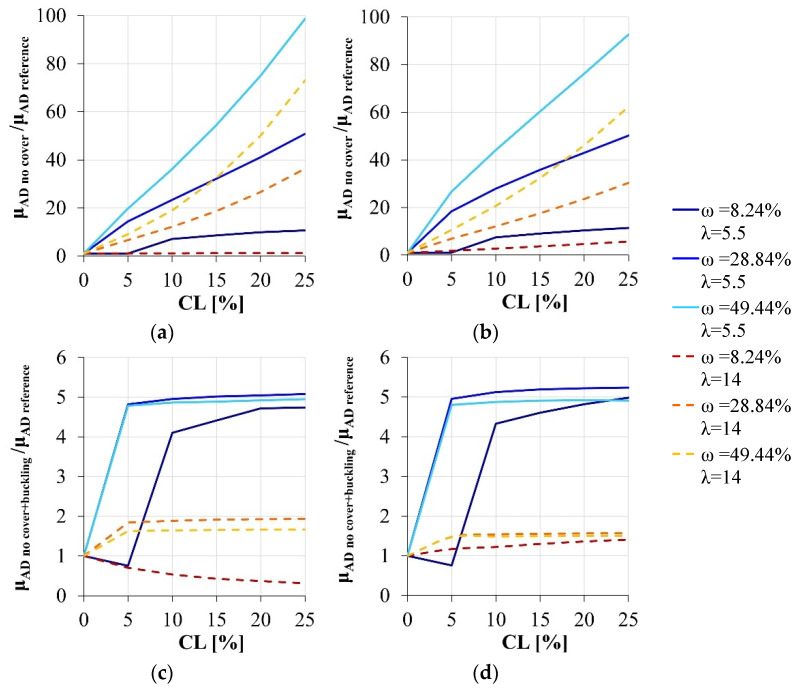
Local ductility variation due to the corrosion degradation: comparison between the simplified models and the reference section. (**a**) ν = 25.65%, section modeled disregarding the concrete cover *vs.* reference section. (**b**) ν = 15.39%, section modeled disregarding the concrete cover *vs.* reference section. (**c**) ν = 25.65%, section modeled disregarding both the concrete cover and the buckling effects *vs.* reference section. (**d**) ν = 15.39%, section modeled disregarding both the concrete cover and the buckling effects *vs.* reference section.

Finally, the feasibility of simplified models in simulating the sectional behavior also in terms of local ductility is analyzed. As highlighted in Figure 9, unlike the nondimensional ultimate bending moment, the adoption of the classical simplified models results in a serious error of the sectional response, thereby threatening the reliability of the entire structural model if the seismic assessment of a corroded structure is researched.

## 4. Discussion and Conclusions

In this paper, a parametric analysis is carried out with the main aim to evaluate the most effective modelling strategy in simulating the corrosion effects at a structural level. The sectional behavior of a corroded rectangular reference section, subjected to different levels of axial load and characterized by a variable amount of longitudinal reinforcement and stirrups, is investigated. Both the effects of the confinement and the lateral instability of the longitudinal reinforcement are considered, by varying the material constitutive laws proposed in the scientific literature. The parametric analysis involved the development of proper moment–curvature relationships, derived according to the classical formulations (congruence and equilibrium equations) in which all the modality of collapse (including the spalling) are accounted for.

The obtained results show that load-carrying capacity depends on: (i) the assumed level for the axial load ($\nu =\frac{N}{b\cdot d\cdot f_{ck}}$), (ii) the stirrups amount, and (iii) the buckling occurrence. Progressively reducing the superimposed axial load level, the stirrups’ contribution becomes negligible. If a simplified model disregarding both the concrete cover and the buckling is adopted, the error committed for the corroded section can be considered tolerable in terms of the load-carrying capacity if the section is subjected to an intermediate superimposed axial load (about the 30% of its sectional capacity). Instead, both the spalling occurrence and the buckling significantly increase the local ductility.

Due to this reason, depending on the typology of structural assessment to perform, different modelling strategies should be adopted. If only the residual load-carrying capacity is researched, a simplified model disregarding the presence of the concrete cover, as well as the confinement and the buckling occurrence, could be adopted. On the contrary, if in the analysis the knowledge of the local ductility is requested, i.e., in case of seismic assessment, a more accurate model should be defined, in which all the main corrosion effects are accounted for (concrete constitutive law variation due to both cover cracking and concrete confinement variation, and steel constitutive law variation accounting for the buckling of the longitudinal reinforcements). Finally, as highlighted by the comparison with some experimental results, it is worth underlining that the local response well catches the structural response of corroded reinforcement concrete elements, provided that excessive slips between reinforcing bars and concrete, i.e., due to fixed-end rotation effects, are avoided. Where this last condition is not fulfilled, more accurate theoretical or numerical models accounting for the occurrence of reinforcement slip in the tensile stage should be considered.

## Data Availability

The raw/processed data required to reproduce these findings cannot be shared at this time as the data also form part of an ongoing study.
